# The Role of Perceived Social Support and Stress in the Relationship between Hope and Depression among Chinese Shadow Education Tutors: A Serial Mediation Model

**DOI:** 10.3390/ijerph19063348

**Published:** 2022-03-12

**Authors:** Mohan Zhang, Yunpeng Wu, Chengjun Ji, Jianfen Wu

**Affiliations:** 1 Jing Hengyi School of Education, Hangzhou Normal University, Hangzhou 311121, China; zmh@hznu.edu.cn (M.Z.); ppwu70@hznu.edu.cn (J.W.); 2 School of Teacher Education, Dezhou University, Dezhou 253023, China

**Keywords:** hope, social support, perceived stress, depression, mediation

## Abstract

The association between hope and depression has been studied, leaving the underlying mechanism of how hope might predict depression unexplored. With a cross-sectional design, this study investigated two possible mediating factors in the relationship between hope and depression among Chinese shadow education tutors, who confront a high turnover rate and are at high risk for depression. Altogether, 221 tutors participated in the survey, and reported their dispositional hope, perceived social support (PSS), perceived stress (PS), and depression. Results indicated that both PSS and PS mediated the relationship between hope and depression. Results also supported the hypothesized serial mediating effect. In other words, hope as a positive disposition may promote PSS, which can mitigate PS. The reduced PS, in turn, alleviates depression. This finding not only shed light on the independent and accumulative mediating effects of PSS and PS, but also has implications for preventive interventions among Chinese shadow education tutors experiencing the enormous pressure of instability. This serial mediation model should be confirmed by further longitudinal study.

## 1. Introduction

Shadow education refers to educational activities outside of formal schooling and is designed to enhance the student’s formal school career [1]. Shadow education usually aims at enhancing primary and secondary students’ academic achievement and has been strongly visible in the world since the turn of the 20th century, especially in China, where high-stakes examinations prevail. The expansion of tutoring trades attracts many thousands of employees working in shadow education institutions (SEI). According to the Ministry of Education of China, 39,516 Chinese private training institutions employed 233,936 tutors in 2020 [2]. Like teachers in other private schools, SEI tutors are confused about the prospects of career development, suffer from lacking social guarantees, low social status, and lack stability due to shadow education institutions’ private property. Additionally, they have a low evaluation of their vocation [3], which may cause great pressure on tutors in SEI. Shadow education has focused on preparation in its curricular content, thus consuming massive amounts of time and money, and increasing students’ academic burden and parents’ household expenditures. Governments worldwide view shadow education as harmful to educational, economic, and social equity. Studies argued that countries should devote much effort to discouraging the negative societal implications [4,5].

Over the past few decades, the shadow education industry in China has been a target of public and official criticism due to its side effects such as fueling the rapid expansion of for-profit training schools, increasing family’s costs on extracurricular classes, exacerbating educational inequalities, and nurturing a generation of examination-oriented pupils. The Chinese government even tried to regulate private tutoring in shadow education by focusing on specific problems. For instance, the Chinese Ministry of Education issued a policy to prohibit in-service school teachers acting as shadow education tutors to organize or provide private tutoring in 2015. In 2021, China released the “Opinions on Further Reducing the Burden of Compulsory Education Students’ Homework and Out-of School Training”. This new regulation is better known as the “double reduction” policy, which has put the full weight of the government behind regulating tutoring and training schools and has strictly regulated shadow education institutions from providing training in core curriculum subjects. Shadow education has become a quite focused problem, while studies usually regarding shadow education from the perspectives of governments, students and parents, little to no research concerns the tutors in shadow education institutions. Teachers are emotional workers, and the teaching profession is categorized as an occupation that has a high impact on emotional health such as depression, stress, and job insecurity [6,7].

Although hope has been considered a protective factor, little is known about the psychological mechanisms that underlie the relationship between hope and depression. Furthermore, it is unclear what potential mediating variables can account for the relationship between hope and depression. In predicting stress-related symptoms, the importance of both perceived stress and coping resources was highlighted [8]. Thus, two psychosocial factors that influence mental health outcomes, perceived stress and perceived social support, were investigated in the current study. The main purpose of the present study is to investigate the serial mediating effects of perceived social support and stress on the association between hope and depression. The following section starts by reviewing the relationship between hope and depression. It is then followed by a discussion of the respective mediating role of social support and stress in the linkage. Finally, the relationship between social support and stress is evaluated to offer support to the hypothesized serial mediation effects. This study pays attention to SEI tutors who confront much more occupational stresses than teachers in public schools, and studies the prevalence of depression and the associated factors contributing to it in order to providing empirical evidence for the healthy development of Chinese shadow education in the new context.

### 1.1. The Relationship between Hope and Depression

Hope is the perceived capability to produce pathways to desired goals and motivate oneself to use these pathways [9]. The hope theory assumes that all individuals are believed to be guided by efforts to reach and obtain goals [10]. As a general disposition to engage in conscious efforts to obtain a goal, hope is expected to extend a range of psychological benefits [11,12]. As for the psychological structure of hope, international studies have identified the same two elements of hope in different cultures: pathways and agency [13,14]. Furthermore, in various studies, hope has been found to play an important role in adaptation to a challenging reality [15,16].

Prior research indicates that hope represents an adaptive personality construct in adult populations. For example, a positive association has been identified between hope and measures of positive psychological adjustment (e.g., life satisfaction) [17,18]. Similarly, a reliable negative association has been identified between hope and measures of negative psychological adjustment (e.g., depressive symptoms) [19,20,21]. As for the focused variable, depression, the inverse relation between hope and depression has been found in middle school students [22], as well as clinical populations, such as psychotherapy outpatients [23] and post-traumatic groups [24]. In addition, a longitudinal study found the prospective effects of hope on depression among college students [25]. In this study, examination of the two cross-lagged panel models supported the negative effects of hope on depression while it did not support the longitudinal effects of depression on hope, which offered solid support for the connection between hope and depression. The present study may contribute to the literature by investigating hope and depression among tutors in shadow education institutions.

Depression, as one of the top public health concerns [26], is one of the most common mental health conditions in the general population, with a prevalence estimated to be between 0.4 and 15.7% across countries [27]. Depression limits an individual’s psychosocial functioning, diminishes their quality of life, and is frequently associated with substantial burdens and costs for the individuals, families, and society [28]. Thus, efforts to develop effective depression prevention and treatment strategies should be made.

Hope demonstrated negative associations with depression; therefore, one possible psychological proceeding is an approach based on hope [29,30]. Volumes of previous studies about shadow education pay close attention to students and parents; tutors’ emotional and psychological status remains unknown. Focusing on Chinese tutors, this study tested a theoretical model in which the relationship between hope and depression is mediated by perceived social support and perceived stress.

To explore depressive symptoms and the underlying vulnerability to depression, scholars have often focused on the cognitive process [31,32,33,34]. According to cognitive theories on depression [35], the maladaptive schemas concerning many life areas lead to a preferential processing bias for schema-congruent information and a consequent dominance of negative or threat-related thoughts, images, and interpretations. Hopeful thinking provides a positive interpretation of oneself, the world, and the future [31] and might play an important role in reducing depression. Based on the above theories and empirical findings, it is reasonable to propose that hope negatively predicts depression (Hypothesis 1).

### 1.2. The Mediating Role of Perceived Social Support

One potential mediator of the relationship between hope and depression is perceived social support. Personality dispositions have been linked to PSS [36,37]. Social support is considered an important coping resource during times of crisis. Perceived social support has been assumed as an important concept for promoting success, positive self-image, and adaptability [38]. The premise of this concept is that confidence in close and meaningful interpersonal relationships is an important resource for both personal and socioemotional development in various life stages [39]. Social support has been identified as essential for reducing depression and related negative outcomes [40,41,42]. Meanwhile, the association between hope and social support has been established in prior studies. For example, hope and perceived social support were positively related among Chinese children with ADHD [43], and hope and social support were related to the ability to persist in college [44]. In addition, hope and social support were identified as an effective adjustment mechanism of resilience to racial discrimination [45].

Thus, we proposed Hypothesis 2: Perceived social support mediates the relationship between hope and depression.

### 1.3. The Mediating Role of Perceived Stress

In recent years, perceived stress has been viewed as a potentially modifiable risk factor for depression [46]. Perceived stress, defined as the extent to which certain life situations are appraised as being stressful to the individual experiencing it [47], can subsequently affect various aspects of one’s life, such as exacerbating negative physical or physiological outcomes [48]. According to the theory on unraveling determinants and consequences of stress [49], perceived stress mediates the relation between personal factors and stress consequences. A cross-sectional study supported the mediating role of stress on the relationship between hope and fatigue [50]. In addition, the effect of hope therapy on stress and depression was supported in an 8-week clinical trial study [51]. Meanwhile, excessive stress beyond one’s ability to adequately cope may further exacerbate psychological fatigue and depressive symptoms [52]. Thus, we might expect that perceived stress would mediate the association between hope and depression.

It should be noted that, in previous studies, hope has also been regarded in terms of elements of psychological capital and tested as moderators of the relationship between stress (and related variables) and negative outcome such as depression [53,54]. However, the current study viewed hope as a personality construct, and personality construct captures relatively stable patterns of thought, emotion, motivation and behavior, and influences perceptions, attitudes and values, as well as reactions to people and situations [55]. Hope was treated as a personality construct in the current study and the perceived stress, in regard to its definition, can be viewed as an individual’s reaction to a situation [47]. This increased the plausibility of a mediation model. Thus, we posit Hypothesis 3: Perceived stress mediates the relationship between hope and depression.

### 1.4. The Relationship between Perceived Social Support and Perceived Stress

As reviewed above, both perceived social support and perceived stress are associated with depression. This suggests that perceived social support and perceived stress might influence each other and then contribute to depression. If that is the case, there are two potential mediating effects that should be taken into account. One possibility is that perceived stress increases depression through social support. The other possibility is that perceived social support decreases stress, which, in turn, decreases depression. The latter seems plausible because researchers have found that perceived social support mediates the relationship between adaptive factors and perceived stress [56].

It should be noted that perceived social support has also been tested as a moderator of the relationship between stress (and related variables) and negative outcomes such as depression [57,58,59]. However, the mediation model was tested in the current study for two reasons. First, the association between social support and stress has been supported in prior studies. Perceived social support has been identified as essential for resilience to stress [60,61]. Second, the sequential mediation effects of perceived social support and perceived stress on the relations between emotional intelligence and wellbeing have been supported in a previous study [62]. Thus, the current study hypothesized that perceived social support and perceived stress mediate the relationship between hope and depression through their chain-mediating effect (Hypothesis 4).

### 1.5. Current Study

**Figure 1:**
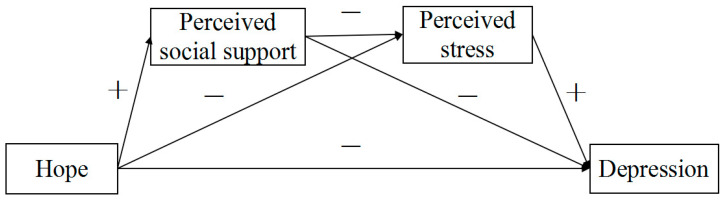
The proposed model.

Based on the current literature, the purpose of the current study was to investigate the relationship between hope and depression among Chinese SEI tutor populations and its underlying mechanism. We proposed a model (see Figure 1). We attempted to answer whether (a) social support and perceived stress mediated the association between hope and depression, respectively, and (b) hope related to depression through the serial mediation of social support and perceived stress.

To answer those questions, we collected cross-sectional survey data from a group of Chinese SEI tutors. The ultimate goal of this study was to provide evidence for effective interventions among SEI tutors in China. In addition, the findings will provide new perspectives for interventions against depression in terms of improving the effectiveness of hope and social support interventions through reducing perceived stress.

## 2. Methods

### 2.1. Participants and Procedures

Participants in this study involved 221 SEI tutors (152 females, 69%) from Hangzhou, the capital city of Zhejiang Province and one of the most important metropolises in China. The participants were sampled through a convenience sampling method using an online advertisement. The advertisement connected participants who were interested to an external page with information and consent to participate in this study. The informed consent form showed yes or no options. Participants who selected “yes” accessed the survey page, and those who selected “no” exited the survey. Participants who chose “yes” were asked to sign and post the written informed consent to the researcher. The period of data collection lasted 14 days in November 2021. The purpose of the study and the autonomy of SEI tutors were highlighted before the survey. Participants were free to withdraw from the survey at any time. Privacy of the participants was guaranteed. Once recruited and consented, the participants completed the survey through the Wenjuanxing platform, the largest online survey tool in China. The survey was conducted anonymously to ensure honest responses and ensure full respect and protection of individual privacy rights before, during, and after the data collection process. The study was approved by the Ethics Committee of Jing Hengyi School of Education, Hangzhou Normal University.

### 2.2. Measures

#### 2.2.1. Hope

The Chinese version of the Adult Dispositional Hope Scale (ADHS) [63] is a 12-item self-report questionnaire used to assess the hope level, which includes agencies (4 items, e.g., “I meet the goals that I set for myself”) and pathway (4 items, “I can think of many ways to get out of a jam”). Each item is rated on a four-point Likert scale, ranging from 1 (completely disagree) to 4 (completely agree). All the responses are based on feelings in general. The total score can range from 8 to 32. A higher score indicates a higher degree of sense of hope. In the present study, Cronbach’s alpha was 0.78.

#### 2.2.2. Perceived Social Support

Perceived social support was measured by the Perceived Social Support Scale (PSSS) [64], validated in the Chinese context previously by Chou [65], showing adequate concurrent and construct validity. The PSSS is a 12-item self-report scale that assesses perceived support arising from three dimensions, namely, family support (4 items, e.g., “I get the emotional help and support I need from my family”), friend support (4 items, e.g., “I can count on my friends when things go wrong”), and other support (4 items, e.g., “There is a special person in my life who cares about my feelings”). Each item emphasizes that all the responses are based on feelings in the last month. Each item is scored on a seven-point scale ranging from 1 (completely disagree) to 7 (completely agree). Thus, total scores can range from 12 to 84, which were applied as indicators of perceived social support, with higher scores indicating greater perceived social support. In the present study, Cronbach’s alpha was 0.97.

#### 2.2.3. Perceived Stress

The Chinese Perceived Stress Scale (CPSS) was used to measure perceived stress. It has widely been used in the mental health assessment of the occupational population. It was revised by Yang et al. (2003) according to the non-Chinese version of the perceived stress scale (PSS) into the Chinese version [66]. The scale has high homogeneity and internal consistency among different populations in China, and Cronbach’s alpha coefficient is 0.797 [67]. The scale consists of 14 items reflecting stress tension (7 items, e.g., “been upset because of something that happened unexpectedly”) and loss of control (7 items, reverse-scored, e.g., “dealt successfully with irritating life hassle”). Participants are required to answer according to their own feelings in the last month on a five-point scale that ranges from 1 (never) to 5 (always). Total scores, which can range from 14 to 70, were applied as indicators of perceived stress in the current study, with a higher score indicating a higher level of perceived stress. In the current study, Cronbach’s alpha was 0.87.

#### 2.2.4. Depressive Symptoms

The Center for Epidemiological Studies-Depression Scale (CES-D) [68] is widely used to measure depressive symptoms. The validated short form of the Chinese version [69] was used in the present study. It is a 10-item self-report questionnaire (e.g., “I was bothered by things that don’t usually bother me”), using a four-point Likert scale ranging from 0 (rarely) to 3 (sometimes); each item emphasizes that all responses are based on feelings in the last week. After two items were reverse-coded, total scores ranged from 0 to 30, with a higher score indicating a higher level of depressive symptoms. Cronbach’s alpha was 0.93 in the current sample.

### 2.3. Statistical Analysis

All data were analyzed using SPSS 23.0 (IBM, Chicago, IL, USA). Categorical data were reported as frequencies; continuous data were reported as mean values. The mediating effect was tested using Hayes’ [70] SPSS macro PROCESS (Model 6). The bootstrapping method with robust standard errors was applied to test the significance of the effects [59]. The bootstrapping method produced 95% bias-corrected confidence intervals (CIs) of these effects from 5000 resamples of the data. If CIs did not include zero, the effects in Model 6 were significant at α = 0.05. All statistical tests were two-tailed.

## 3. Results

### 3.1. Characteristics of Participants

Sample characteristics data from 221 SEI tutors were included in the analysis (Table 1). The age groups of the participants were distributed as follows: 25 years and below (*n* = 38, 17%), 26–35 years (*n* = 130, 59%), 36–45 years (*n* = 38, 17%), 46 years and above (*n* = 15, 7%). For education background, most of the participants (*n* = 164, 74%) reported holding a bachelor’s degree, 20 participants (9%) held a master’s degree or above, 37 participants (17%) had completed higher vocational college and below education. For work experience, most of the participants (*n* = 120, 54%) worked in SEI for 1–5 years, 26 participants (12%) worked in SEI no more than 1 year, 42 participants (19%) worked in SEI for 6–10 years, and 33 participants (15%) worked in SEI more than 10 years. As for the monthly income, 68 participants’ income (31%) were no more than CNY 4999, 99 (45%) were between CNY 5000 and 9000, 34 (15%) were between CNY 10,000 and 14,999, and 20 (9%) were more than CNY 15,000. The participants were asked to describe the scope of their institution’s business. In summary, 81 participants (37%) reported that the scope of their institution’s business is academic subject tutoring; 90 participants (41%) reported that the scope of their institution’s business includes academic subject tutoring and non-academic tutoring, but mainly academic subject tutoring; 27 participants (12%) reported that the scope of their institution’s business includes academic subject tutoring and non-academic tutoring, but mainly non-academic subject tutoring; and 23 participants (10%) reported that the scope of their institution’s business is non-academic tutoring.

### 3.2. Descriptive Statistics and Correlations

The four questionnaires were tested for common method biases with Harman’s single-factor test. Through exploratory factor analysis, eight factors with eigenvalues over 1 were obtained. The first factor accounted for 18.58% of the total variance, which was far below the threshold (i.e., 40% of the explained variance). This demonstrated that the common method biases were not significant.

As shown in Table 2, hope was significantly and positively associated with social support, and negatively associated with perceived stress and depression; social support was significantly and negatively associated with perceived stress and depression; and perceived stress was significantly and positively associated with depression.

### 3.3. Testing for the Mediation Effects

First, we conducted the regression analyses before testing the mediation model to explore the paths between the study variables. All these correlated variables were included in the regression analyses. As shown in Table 3, the results of the total sample confirmed that: (1) the total effect of hope on depression was significant (*β* = 0.510, *p* < 0.001), and after the mediators entered the regression, the direct effect of hope on depression became insignificant (*β* = −0.028, *p* = 0.653); (2) hope positively predicted perceived social support (*β* = 0.546, *p* < 0.001), and negatively predicted perceived stress (*β* = −0.468, *p* < 0.001); (3) perceived social support negatively predicted perceived stress (*β* = −0.325, *p* < 0.001); (4) perceived social support negatively predicted depression (*β* = −0.168, *p* < 0.01); and (5) perceived stress positively predicted depression (*β* = 0.604, *p* < 0.001).

Next, serial mediation analysis using the Bootstrap (model 6, sampling 5000 time) method was conducted to examine the indirect effects of hope on depression. The results were all significant (95% CI did not include 0). As shown in Table 4, the results indicated that the total indirect effect of hope on depression in the full model was significant −0.482, which was significant with a 95% CI [−0.612, −0.360]. Specifically speaking, (1) the indirect effect of hope → social support → depression was significant (effect = −0.092, 95% CI [−0.180, −0.011]); (2) the indirect effect of hope → perceived stress → depression was significant (effect = −0.283, 95% CI [−0.389, −0.194]); and (3) the indirect effect of hope → social support→ perceived stress → depression was also significant (effect = −0.107, 95% CI [−0.149, −0.065]).

**Figure 2:**
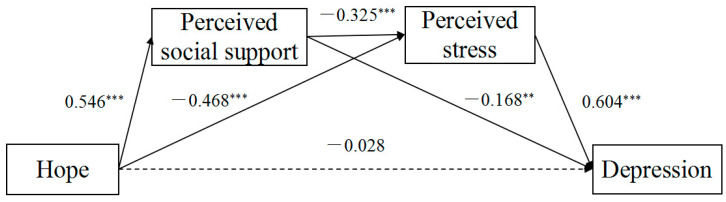
Associations between hope and depression for the whole sample (*N* = 221). Note. Coefficients are standardized. ** *p* < 0.01, *** *p* < 0.001.

Those findings indicated that social support and perceived stress mediated the association between hope and depression both, respectively, through the chain intermediary of social support and perceived stress. The final model for the whole sample is shown in Figure 2.

## 4. Discussion

Taking a vulnerable population (i.e., Chinese SEI tutors have a high turnover rate and unclear career prospects) as participants, the current study extended the literature by demonstrating the role of perceived social support and perceived stress in mediating the association between hope and depression both, respectively, and through their sequential mediation effect. The evidence is found in favor of three hypothesized indirect effects: (1) hope → PSS → depression, (2) hope → PS → depression, and (3) hope → PSS → PS → depression.

### 4.1. Hope and Depression

The current study found that hope was negatively correlated with depression, which was consistent with previous studies [23,24,25]. However, contrary to our expectations, the direct predictive effect of hope on depression (Hypothesis 1) was not supported by our results. The results indicated that hope might not affect Chinese SEI tutors’ depression directly, but influence it through their perceived social support and stress. These results also indicated the necessity to explore the underlying mechanisms of the relationship between hope and depression.

### 4.2. Mediating Effect of Perceived Social Support

By analyzing the influence of hope on the depression path through social support, we found that hope may lower the occurrence of depression by improving the perceived social support of SEI tutors.

The mediating effect of social support may be explained as individuals with higher dispositional hope being better able to recognize and have better opportunities to enhance their social support, which, in turn, is associated with a lower level of depression. For SEI tutors with high hopeful thinking, even though the stressful situation seems to be overwhelming, the sense of being supported allows them to cope with it. Social support not only provides the information and guidance necessary to assist in assessing the threat and planning coping strategies [71], but also improves their sense of meaning and purpose [72]. Adequate social support can provide a safe environment for individuals to talk freely about negative experiences and related emotions, thus reducing individual depression symptoms [73]. Relevant empirical studies also indicated that social support could reduce depression [74].

### 4.3. Mediating Effect of Perceived Stress

Results showed that perceived stress mediated the relation between hope and depression, supporting H3. First, adaptive personal resources (e.g., hope) can foster the process of appraising stressful events and maintaining a manageable level of stress [75]. Second, the concept of perceived stress highlights how people feel about stress, not just stress itself. It is an individual’s evaluation of a situation and events beyond their self-ability, that is, a cognitive evaluation of stressful events and situations. For SEI tutors, the strict restriction of the “double-reduce” policy posed stress to them. A stressor typically triggers a stress reaction if that stressor is perceived as a threat or demand or if the coping resources are perceived as insufficient for handling the given situation [66]. Indeed, the perception of stressors as threatening, uncontrollable, or overwhelming is highly predictive of subsequent negative psychological symptoms [76,77]. Finally, as prior research indicated, hopeful thinking could help individuals distract their attention from negative events and promote the adoption of more adaptive strategies to deal with negative events, thus alleviating depression symptoms [23,78].

### 4.4. The Sequential Mediation Effect of Perceived Social Support and Perceived Stress

We proposed that SEI tutors with higher levels of hope may likely experience greater support within the environment, such as from family and friends, contributing to a decreased possibility of stress. We expected that perceived social support and stress would be sequential mediators in the association between hope and depression. Our results supported Hypothesis 4.

The pathway of hope → perceived social support → perceived stress → depression was significant, and the indirect effect of hope on depression via perceived social support and perceived stress was statistically significant. That is, perceived social support and perceived stress mediated the association between hope and depression. According to the theory of social connectedness, social connectivity represented by “keeping a close relationship with society” can meet the individuals’ belonging needs [79] and can reduce their stress. Our result is consistent with prior findings that beyond the direct influences of perceived social support on well-being, social support might significantly influence perceived stress in the prediction of well-being [80,81]. Therefore, during the rush period of policy change, the sense of belonging of SEI tutors with more social support will be better satisfied; in turn, their perceived stress might be reduced.

## 5. Conclusions, Implications, and Future Directions

The findings of the current study serve as a solid foundation for future studies designed to understand the specific mechanisms underlying hope and depression. The study findings indicate that hope, as a positive disposition, can reduce depression in SEI tutors by promoting perceived social support and reducing perceived stress, respectively. Hope also reduces depression in SEI tutors through a chain-mediating effect of social support and perceived stress. Overall, hope can directly foster social support and reduce perceived stress, and perceived social support can decrease perceived stress to protect vulnerable people from depression, which has implications for preventive interventions among shadow education tutors under unprecedented instability.

The results from this study enrich the existing literature that examines the relationship between hope and depression among Chinese SEI tutors. Importantly, our findings on the factors that affect tutors’ depression can help us formulate policies and design corresponding countermeasures to protect tutors’ mental health. This study explores the influencing factors of SEI tutors’ depression, which could provide a reference for promoting the development of the Chinese shadow education industry sustainably in the context of primary and secondary education “burden reduction”. The findings can also guide SEI tutors to further transform hope and sufficient social support into psychological resources facing the future and reduce their perceived stress to actively cope with the difficulties during the career shock period. There are several practical implications based on the suggestions such as adjusting to a less competitive environment, providing psycho-social support, providing hope intervention and improving self-esteem, which can encourage hope and guide SEI tutors to build confidence in the future. It is imperative for public policies to pay attention to SEI tutors’ social welfare for they are one of the most important parts of private education, and provide alternative career pathways for SEI tutors. Shadow education institutions are encouraged to promote SEI tutors’ professional development, building a high-quality teaching profession to meet the requirement of SEI tutors’ long-term career development. SEI tutors could benefit from the hope intervention by reconsidering their personal goals and potential obstacles in the workplace. Acquiring support from family members and peers could prevent SEI tutors from developing stress or depressive mood. SEI tutors are recommended to utilize their social network to improve mental health.

Several limitations of the current study should be noted. First, we used a convenience sample so the findings may not be generalized to all SEI tutors. The oversampling of some characteristics may not be representative of the Chinese SEI tutors population. For example, the years of workplace experiences of SEI tutors have a positive influence on their job success and employment status [82] and, hence, improve their well-being and reduce depression. Future research should focus on the SEI tutors who stay in this industry for more than 5 years and include SEI tutors who are no longer in their job but did not yet find another job. Second, the data were cross-sectional, limiting us from making causal inferences about the associations among the variables. Thus, longitudinal or experimental studies should be conducted to confirm the observed associations and extend the current findings, especially the moderate and mediating relationship between support and stress. Moreover, this study does not adequately control other factors that may influence depression, such as SEI tutors’ income and whether the SEI tutors were in a satisfying relationship. The majority of this population had a low income, especially for junior tutors, and this will cause a lot of stress and increase the risk of depression. Finally, the current study relied on self-report measures. Future studies that include objective measures (e.g., standardized observations and interviews) would be important for a more accurate assessment of the effects of hope on depression.

Future research suggestions may be warranted on this topic, such as, firstly, depression and pressure coping strategies by building up hope and accessing more social support for teachers, especially teachers in private schools. Secondly, future studies should include more situational factors such as burnout, anxiety and so on. Thirdly, studying teachers’ stress and depression from the perspective of teachers’ career development may lead to more findings that are practical.

## Figures and Tables

**Table 1:** Demographic statistics (*N* = 221).

Variables	Frequency (*n*)	Percent (%)
Gender		
Female	152	69
Male	69	31
Age group		
25 years and below	38	17
26–35 years	130	59
36–45 years	38	17
46 years and above	15	7
Education		
higher vocational college and below	37	17
University/Bachelor’s	164	74
Master’s and above	20	9
Work experience		
Less than 1 year	26	12
1–5 years	120	54
6–10 years	42	19
10 years and above	33	15
Monthly income		
CNY 4999 and below	68	31
CNY 5000–9999	99	45
CNY 10,000–14,999	34	15
CNY 15,000 and above	20	9
Scope of institution business		
Academic subject tutoring	81	37
Mainly academic tutoring	90	41
Mainly non-academic tutoring	27	12
Non-academic subject tutoring	23	10

**Table 2:** Means, standard deviations and correlation coefficients for key variables.

	*M*	*SD*	1	2	3	4
1. Hope	21.66	4.34	1			
2. Perceived social support	56.36	15.97	0.546 ***	1		
3. Perceived stress	41.78	8.01	−0.646 ***	−0.581 ***	1	
4. Depression	11.71	8.36	−0.510 ***	−0.534 ***	0.720 ***	1

Note. *** *p* < 0.001.

**Table 3:** Regression analysis results.

Regression		Model Index		Coefficients	
Outcome Variables	Independent Variables	*R* ^2^	*F*	*β*	*t*
Perceived social support	Hope	0.30	92.87 ***	0.546	9.64 ***
Perceived stress	Hope	0.49	105.28 ***	−0.468	−8.13 ***
	Perceived social support			−0.325	−5.64 ***
Depression	Hope	0.54	84.59 ***	−0.028	−0.45
	Perceived social support			−0.168	−2.85 **
	Perceived stress			0.604	9.35 ***

Notes. *N* = 221. *β* = Standardized coefficients. ** *p* < 0.01; *** *p* < 0.001. All variables were standardized.

**Table 4:** Total, direct and indirect effects of hope on depression.

Paths	Full	
	Effect	95% CI
Total effect	−0.510	[−0.625, −0.396]
Direct effects	−0.028	[−0.152, 0.095]
Indirect effect		
Total indirect effects	−0.482	[−0.612, −0.360]
Hope → Social support → Depression	−0.092	[−0.180, −0.011]
Hope → Perceived stress → Depression	−0.283	[−0.389, −0.194]
Hope → Social support→ Perceived stress → Depression	−0.107	[−0.149, −0.065]

Notes. *N* = 221. Bootstrap sample size = 5000. CI = confidence interval.

## Data Availability

According to the data access policies, the data used to support the findings of this study are available from Hangzhou Normal University, upon a reasonable request made by email: zmh@hznu.edu.cn.
